# IgG4-related mastitis managed without excision or steroid therapy

**DOI:** 10.1186/s40792-024-01826-9

**Published:** 2024-02-02

**Authors:** Hirokazu Matsushima, Masayuki Kikuchi, Rika Miyabe, Sota Yamaguchi, Hidenori Kita, Junko Kobayashi, Takashi Ando, Koji Atsuta, Takayoshi Soga, Tsunehiro Shintani

**Affiliations:** 1 Department of Surgery, Japanese Red Cross Shizuoka Hospital, 8-2, Ottemachi, Aoi-Ku, Shizuoka, Shizuoka 420-0853 Japan; 2 Tosen Clinic, 1-20, Gohukucho, Aoi-Ku, Shizuoka, Shizuoka 420-0031 Japan; 3 Department of Rheumatology, Japanese Red Cross Shizuoka Hospital, 8-2, Ottemachi, Aoi-Ku, Shizuoka, Shizuoka 420-0853 Japan

**Keywords:** IgG4-related sclerosing disease, IgG4-related mastitis, Breast mass

## Abstract

IgG4-related mastitis is an extremely rare IgG4-related sclerosing disease involving the breast that must be differentiated from breast cancer. There is currently no consensus regarding the optimal treatment strategies. Here, we report a case of IgG4-related mastitis followed up without excision or steroid therapy. Although the association between IgG4-related mastitis and breast cancer remains unclear, regular follow-up imaging and measurement of serum concentrations of disease activity markers may allow for follow-up without excision or steroid therapy.

To the editor,

We read the recent case reports on systemic steroid therapy for IgG4-related mastitis by Tsuda [1] and Itakura [2]. IgG4-related mastitis (IgG4-RM), an extremely rare IgG4-related sclerosing disease (IgG4-RD) similar to breast cancer, has no defined treatment strategy. However, excision or steroid therapy is used in most cases. We describe a case of IgG4-RM wherein stable disease was achieved without excision or steroid therapy.

A 47-year-old woman with no medical history complained of swelling in both submandibular areas. Blood tests revealed IgG4 of 422 mg/dL. Submandibular gland biopsy allowed a diagnosis of IgG4-RD. Endometrial polyps were excised, revealing no malignancy. Computed tomography revealed no abnormalities in other organs. As IgG4-RM did not cause organ damage, steroid therapy was not administered, and she was followed up regularly. Four years later, ultrasonography for breast cancer screening showed an irregularly shaped 36-mm hypoechoic mass with an abundant vascular signal in the upper left medial region (Fig. 1A). Magnetic resonance imaging (MRI) showed an irregularly shaped contrast-enhanced mass (Fig. 1C). Pathological examination of needle biopsy revealed a highly IgG4-positive plasma cell infiltration (115 cells/1 HPF), with an IgG4/IgG rate of 63% (Fig. 2). No neoplastic lesions were observed. IgG4-RM was diagnosed based on clinical and pathological findings. She refused excision or steroid therapy. One year and eight months have passed since IgG4-RM diagnosis with regular imaging follow-up, without increase in size (Fig. 1B) or symptoms. Blood tests revealed no obvious elevation in IgG4 levels (470 mg/dL).

**Fig. 1:**
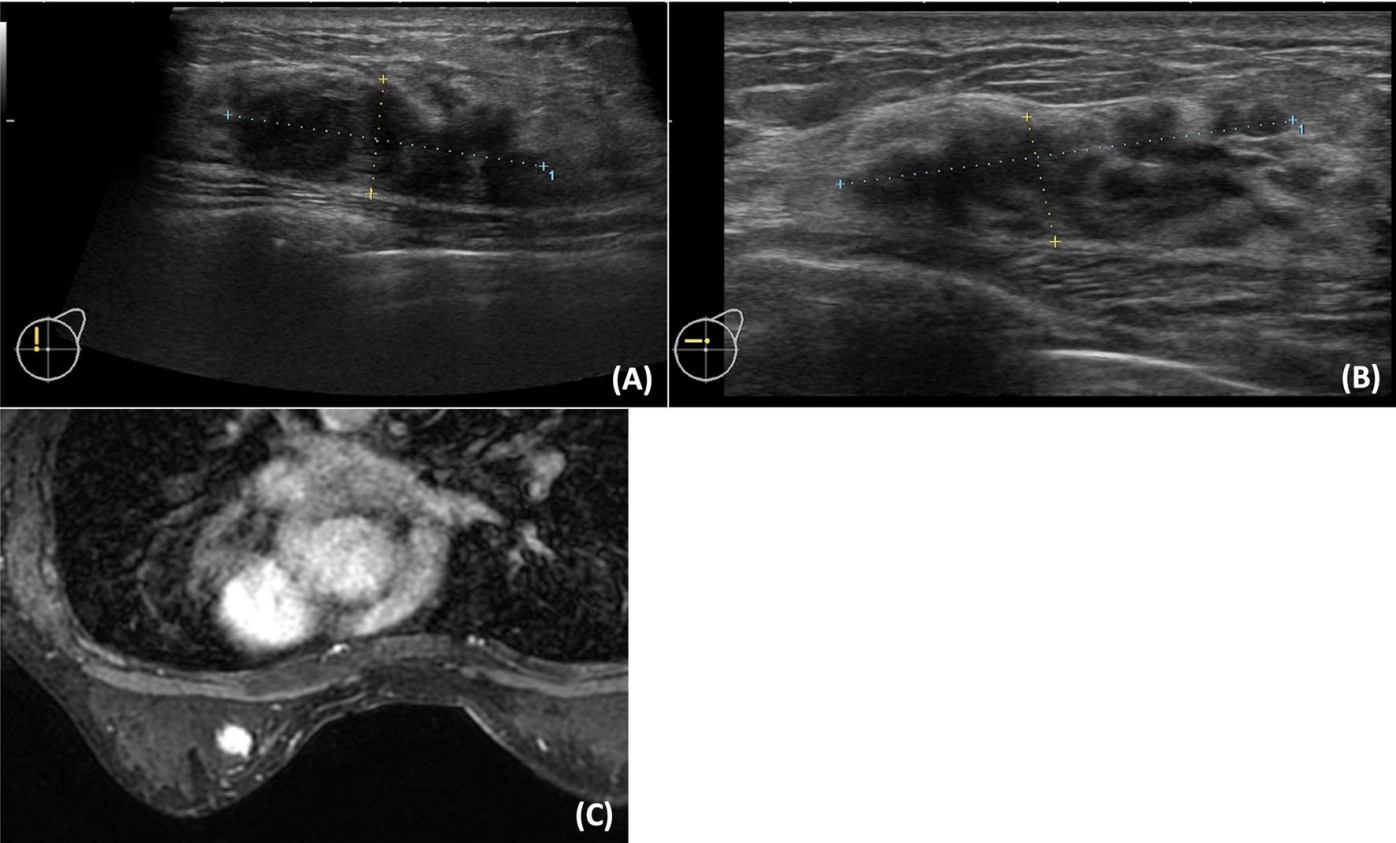
Breast ultrasonography and MRI. **A** A breast ultrasonography showing an irregularly shaped 36-mm low echoic mass with abundant vascular signal in the upper left medial region. **B** Breast ultrasound showing no increase in the mass one year and eight months after the diagnosis of IgG4-RM. **C** Breast MRI showing an irregularly shaped contrast-enhanced mass

**Fig. 2:**
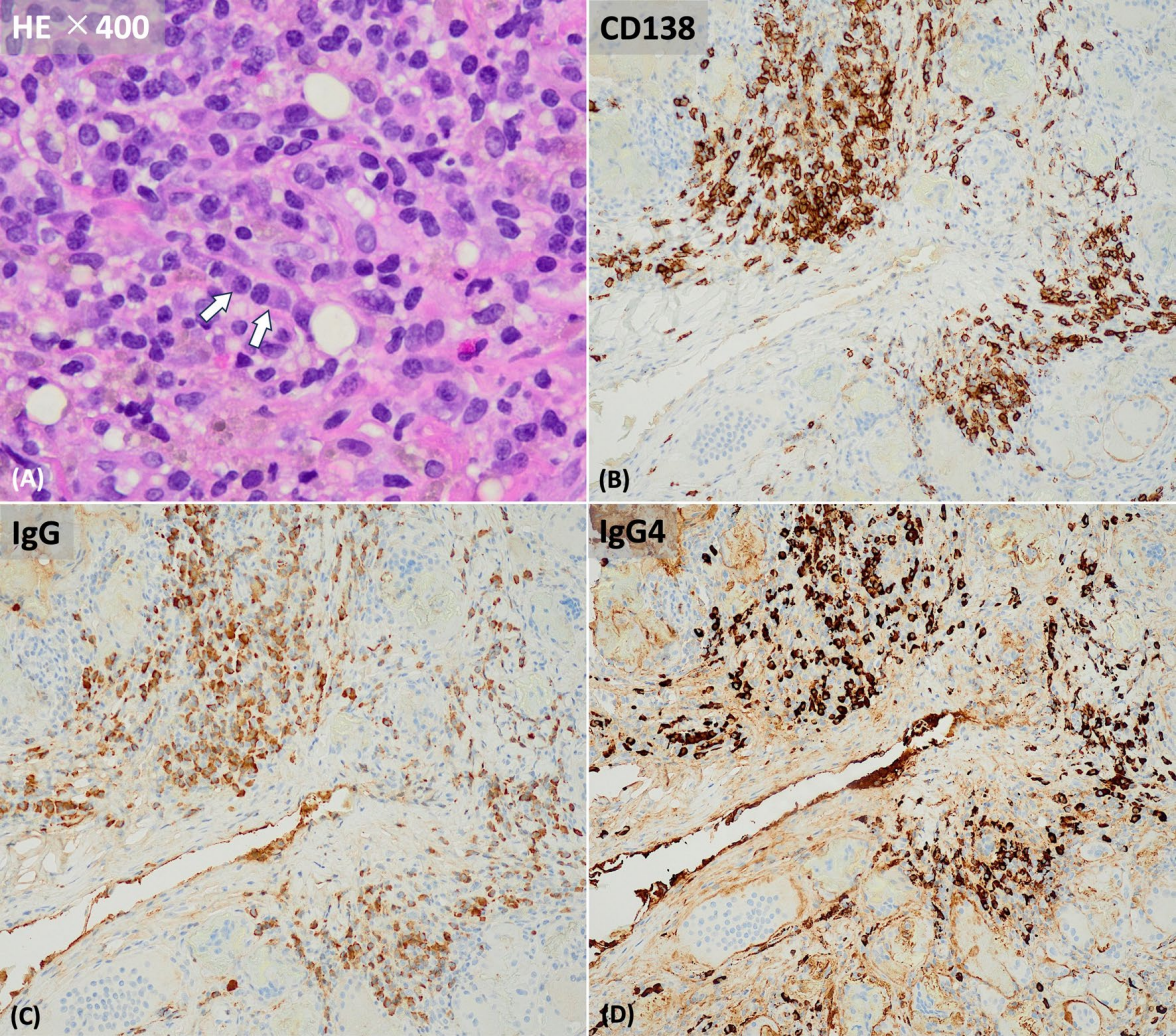
Pathological findings of the biopsy specimen of the breast. **A** This micrograph is taken at 400 × magnification and stained with hematoxylin and eosin (→: plasma cells). **B** CD138 immunostaining showed numerous plasma cell infiltrates. **C, D** Infiltration of several plasma cells is noted. Pathological findings reveal high IgG4-positive plasma cell infiltration (115 cells/1 HPF). IgG-positive plasma cell was 182 cells/1 HPF, and the IgG4/IgG rate is 63% (115/182)

Previously reported cases of IgG4-RM were treated with excision or steroid therapy [1–16]. Most cases have been treated with either excision or steroid therapy, but no recurrence at 12 months has been reported; furthermore, no study has reported the combined use of excision and steroid therapy. The steroid therapy shown to be effective; however, side effects include weight gain, altered appearance, moon face, depression, fatigue, mood swings, increased hair loss, sleeplessness, and stomach discomfort [17]. IgG4-RM is most common in middle-aged women [12, 15], who are often reluctant to take oral steroids because of these side effects [17]. Association between IgG4-RM and breast cancer remains unclear, and because most cases are painless [12, 15], excision and steroid therapy may be unnecessary. Contrastingly, Asano et al. reported an association between IgG4-RD and malignancy, finding a higher incidence of malignancy in the first 12 years after IgG4-RD diagnosis than in the general population. They reported a high association with malignancy with a standard incidence ratio of 3.53 (95% CI 1.23–5.83) within 1 year of diagnosis [18]. Furthermore, among patients with IgG4-RD, serum concentrations of IgG, IgG4, sIL-2R, and circulating immune complex (CIC) at diagnosis were significantly higher in the malignant disease development group [18]. Although IgG4-RM may not require treatment, it should be followed up regularly with imaging and serum concentration evaluations of activity markers (especially during the first year) as the association with breast cancer is unclear. As the serum concentration of IgG4 was followed in this case, if the serum concentration of IgG4 surpasses 749 mg/dL [18] or if the breast mass tends to increase, another biopsy and examination for breast cancer should be performed. Similar to the present case, Cheuk et al. reported a case of IgG4-RM without excision or steroid therapy, with no recurrence in 6 years [4]. Furthermore, regarding the mastitis site or occurrence of IgG4-RD in other organs, no obvious difference was found between the cases treated with resection or steroid therapy and those who were only followed up [1–16].

Non-essential excision can be avoided by considering IgG4-RM as a differential diagnosis for breast masses. Regular follow-up imaging and assessment of disease activity markers may allow management without steroid therapy.

## Data Availability

Not applicable.

## Abbreviations

IgG4-RD: IgG4-related sclerosing disease
IgG4-RM: IgG4-related mastitis
MRI: Magnetic resonance imaging
